# Biotemplated Photocatalytic Micromotors for Effective Inhibition of Bacterial Growth

**DOI:** 10.1021/acs.chemmater.5c00885

**Published:** 2025-08-19

**Authors:** Carmen Cuntín-Abal, Miriam Chávez, Beatriz Jurado-Sánchez, Alberto Escarpa

**Affiliations:** † Department of Analytical Chemistry, Physical Chemistry, and Chemical Engineering, Universidad de Alcala, Alcala de Henares, Madrid E-28802, Spain; ‡ Chemical Research Institute “Andres M. Del Río”, Universidad de Alcala, Alcala de Henares, Madrid E-28871, Spain

## Abstract

Bacterial infections represent a major threat that can cause millions of deaths worldwide, where bacterial species can colonize and grow into highly resistant biofilms. Autonomous micromotor propulsion can improve the overall efficiency of bacterial growth inhibition versus other static processes, leading to novel methods for bacterial treatment. Here, biotemplated magnetic and photocatalytic micromotors are synthesized using *E. coli* and *S. aureus* as bacterial templates with different morphological features, such as shape and size, to obtain reproducible micromotors, followed by decoration with Fe_3_O_4_ nanoparticles for magnetic guidance into biofilms. Then, photoactive BiOCl crystals are grown on the micromotor surface for in situ photocatalytic generation of reactive oxygen species (ROS) for efficient bacterial growth inhibition. Thanks to their high photostability, *E. coli*@Fe_3_O_4_@BiOCl micromotors enabled controlled and efficient ROS production under sterilization conditions by using 375 nm light to trigger an oxygen vacancy generation mechanism within a biocompatible, tailored 3D-printed electrochemical cell. The (photo)­electrochemical ROS generation correlated well with highly efficient bacterial growth inhibition, demonstrating the potential application of the collective dynamics of these multifunctional biotemplate-based micromotors. The concepts described here are promising for the development of future strategies against resistant bacteria by understanding the underlying processes behind them.

## Graphical abstract

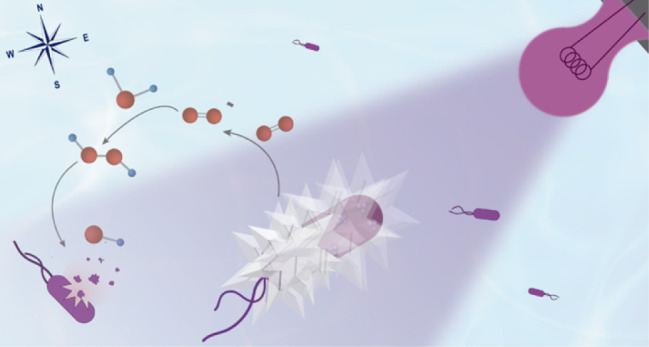

## Introduction

Bacterial infections represent a major threat that can cause millions of deaths worldwide. Such an issue is critical in hospitals, where resistant bacterial species can colonize and grow into highly resistant biofilms on biomedical devices or implants.^1,2^ New means of understanding the underlying processes behind bacterial communication and the effectiveness of inactivation processes are crucial to solving such challenges. Micro/nanomotors are microscale devices that can convert different forms of energy (light, ultrasound, magnetic fields, etc.) into motion.^3–9^ Compared with static processes, micromotors allow for enhanced mixing, leading to dynamic alternatives for bacterial treatment.^10^ For example, catalytic zeolite micromotors with a dual Ag catalytic/killing layer have been used for killing *E. coli* bacteria.^11^ TiO_2_ has been coated onto Mg spheres for light-triggered ROS generation for *Bacillus globigii* spores deactivation.^12^ Among the different strategies, biocompatible magnetic propulsion provides effective guidance to the point of need. Photocatalytic materials such as BiOCl have been combined with magnetic CoNi nanowires for pollutant degradation (Rhodamine B) through the combination of micromotor motion and ROS generation.^13^ Photothermal inactivation of *Klebsiella pneumoniae* has been achieved with magnetically propelled spirulina-coated micromotors.^14^ Silica tubes/magnetotactic bacteria hybrids also have been used for enhanced antibiotic delivery and biofilm killing.^15^

Biotemplate-based micromotors rely on natural structures that provide identical molds for the self-assembly of magnetic or light-responsive materials for fully biocompatible micromotors and large-scale reproducible production. One of the first examples was illustrated by coating spinal vessels from plants with Ti and Ni for magnetic-based propulsion.^16^ Sporopollenin capsules,^17^ natural microalgae,^18,19^ red blood cells,^20,21^ or sperm cells have been also used for this purpose.^22–25^ The use of bacterial cells is also convenient for micromotor fabrication due to their ability to mimic the environment for more active and mimetic biofilm inactivation processes. As such, drug-loaded mesoporous silica microtubes were combined with *Magnetosopirrillum gryphiswalense* bacteria as the propulsion unit integrated into the inner tube, with average velocities of 25 μm/s under the action of electromagnetic coils.^15^ Genetically engineered *E. coli* MG1655 bacteria have also been modified with streptavidin for the assembly of red-blood-cell-derived nanoerythrosomes. The resulting micromotor combines the self-mobility features of bacteria with average speeds of over 14 μm/s.^26^

The monitoring of bacterial biofilm inactivation can be achieved by techniques such as LIVE/DEAD staining, optical density measurements, or impedance monitoring. Yet, in most cases, the studies are performed offline, taking the samples at a fixed time, while bacterial colonization takes place in confined environments. This can influence the whole process, providing, in some cases, uncertain information. As an alternative to these *post-fact* assessments, micromotors can be driven into the bacterial walls and dynamically develop in situ the action required for bacterial inhibition.

Herein, a biotemplate-based multifunctional micromotor approach is adopted by fixation of *E. coli* or *S. aureus* bacteria with glutaraldehyde, followed by the assembly of magnetic Fe_3_O_4_ nanoparticles. The micromotors were then used as units for the controlled crystallization of photoactive BiOCl. The multifunctional micromotors combined light-responsive abilities for ROS generation for inhibition of bacterial growth assisted by magnetically controlled propulsion. To gain more insight into the interaction of bacteria–micromotors and to get the optimal conditions for bacterial inactivation, we have developed a 3D-printed tailored device with a dedicated chamber that allows irradiation with near-ultraviolet (UV) light under controlled ON-OFF actuations. The device also integrates electrodes for monitoring ROS as an assessment of the micromotors’ photocatalytic activity. 3D printing technology has enabled the production of tailored, mass-scale devices and platforms with a myriad of materials^27^ that are useful in a variety of fields,^28^ including the monitoring of bacterial biofilm growth.^29,30^ The new analytical approach described here holds considerable promise for the control and development of future strategies against resistant bacteria and the underlying processes behind it.

## Experimental Section

### Synthesis of Fe_3_O_4_ Nanoparticles

The Fe_3_O_4_ nanoparticles were synthesized using a coprecipitation method. First of all, 50 mL of 8 mM FeCl_3_ (cat. 157740, Sigma-Aldrich, Spain) and 10 mM FeCl_2_ (cat. 372870, Sigma-Aldrich, Spain) solutions were prepared using distilled water (DI) and placed under vigorous stirring in a beaker until dissolved. After that, 10 mL of NaOH (cat. 106498, Merck, Spain) 0.5 M was added dropwise. The resultant Fe_3_O_4_ nanoparticle solution was ultrasonicated for 20 min (pulse 2–1, amplitude 80%) and washed by centrifugation, then resuspended in 50 mL of DI water. Finally, 100 μL of HCl 5 M (cat. 320331, Sigma-Aldrich, Spain) was added to the solution and stored at 5 °C for further use.

### Bacterium Culture and Bacteria@Fe_3_O_4_ Biotemplate Micromotor Synthesis

First, 1 mL of *E. coli* cells (cat. EC11303, Sigma-Aldrich, Spain) was grown in 50 mL of Luria–Bertani (LB) broth overnight at 37 °C and 350 rpm in a conical tube. Next, the bacterial solution was washed with DI water 3 times by centrifugation (3000 rpm, 5 min), and the resultant bacterial pellet was stabilized by immersion in a 2.5% glutaraldehyde solution (cat. A17876.AP, Thermofisher, Spain) for 6 h under external agitation (500 rpm). After that time, the stabilized bacterium was washed 3 times by centrifugation and redissolved in 50 mL of Fe_3_O_4_ nanoparticles (previously ultrasonicated for 20 min). The solution was incubated overnight under external agitation at 500 rpm in a conical tube. Finally, the Fe_3_O_4_ nanoparticle-bound bacteria were washed three times using a neodymium magnet (NdFeB) magnet and resuspended in 20 mL of DI water. The procedure is analogous to the case of *S. aureus* but using the corresponding bacterial cells (catalog no. 6571, Merck Millipore, Spain).

### Synthesis and Characterization of Bacteria@Fe_3_O_4_@BiOCl-Biotemplate Micromotors

The bacteria@Fe_3_O_4_ assembly obtained previously were retained by the NdFeB magnet and dried. After that, 60 mL of a solution 0.02 M of bismuth­(III) nitrate pentahydrate (cat. 248592, Sigma-Aldrich, Spain) previously ultrasonicated for 20 min was added to the solution and mixed. The solution was separated into 3 beakers (solutions A–C), each containing 20 mL of the solution. To obtain the different crystallizations, all the solutions were subjected to vigorous stirring, and 1 mL of acetic acid (cat. ARK2183, SAFC, Spain) was added to solution A, 1 mL of ethylene glycol (cat. BP230–1, Fisher Bioreagents, Spain) was added to solution B, and 1 mL of glycerol (cat. BP229–1, Fisher Bioreagents, Spain) was added to solution C. After that, 6 mL of a sodium chloride 0.06 M solution (cat. 231–598–3, Fisher Scientific, Spain) was added to solutions B and C. To solution A, 6 mL of a sodium chloride 0.06 M/sodium acetate 0.12 M solution (cat. 62681000, Merck, Spain) was added. All the solutions were mixed for 2 h using a stirring plate. After that period, the solutions remain static for 1 h. The resultant solutions, containing the bacteria@Fe_3_O_4_@BiOCl-biotemplate micromotors, were washed 3 times with DI water using a NdFeB magnet. Finally, the solutions were dropped in a plastic Petri dish and allowed to dry overnight at room temperature. The next day, the dry micromotors were dissolved in PBS 0.01 M, adjusting the concentration to 1 mg/mL.

Scanning electron microscopy (SEM) characterization of the micromotors was performed on a JEOL JSM-6335F microscope coupled to an XFlash detector 4010 (Bruker). X-ray photoelectron spectroscopy (XPS) characterization was performed with a SPECS GmbH-UHV system coupled to a PHOIBOS 150 9MCD analyzer. Spectra analysis and fitting was conducted with Kherve Fitting software.

### Magnetic Device and Micromotor Propulsion

Micromotor motion was achieved using a 3D-printed device with an electromagnet consisting of a copper solenoid connected to an adjustable power supply. An Arduino board was used to control the application of variable voltages to control the speed (1.5 and 4 V), allowing for the modulation of the speed of the magnetic micromotors. The magnitude of this magnetic field was obtained empirically by measuring it on the electromagnet surface using a tailor-made device. After that, the magnetic field at the position of the micromotor was calculated. Videos of the micromotors were taken with an inverted Nikon Eclipse Ti–S/L100 optical microscope coupled with a Zyla sCMOS camera.

### 3D-Printed Electrochemical Cell Design

The design of the measurement platform, including the fully 3D-printed electrochemical cell, was performed with computer-aided design (CAD) software (Student License, Autodesk, United States). PrusaSlicer was the software employed to prepare the .STL files. Considering the design of the device proposed, the following parameters were selected: 0.2 mm layer height, 100% infill percentage, 1.1 extrusion multiplier, 230 °C nozzle temperature, 90 °C bed temperature, and 25 mm/s print speed. Both filaments, poly­(ethylene terephthalate glycol) (PETg) and poly­(lactic acid) doped carbon black (PLA-CB), were kept in a filament dryer at 45 °C during the printing process to avoid the adsorption of moisture. Filaments were manually changed when necessary. The electrochemical cell comprised three electrodes with full dimensions of 0.4 mm thick and slightly variable width and length (working electrode, WE: 2 mm wide, 12.5 mm long; reference electrode, RE, counter electrode, CE: 1 mm wide, 13.0 mm long), showing a pitch separation of 3 mm in a microband configuration. The electrode area exposed to the solution is 1.7 mm × 2 mm (3.46 mm^2^) for the WE, and 1.73 mm^2^ for the CE and RE. The electrode configuration (minimizing the distance between the electrodes) was selected to reduce the ohmic drop contribution. The electrochemical device was printed using an Original Prusa i3MK3S+ printer (Prusa Research, Czech Republic) with a 0.4 mm nozzle. The rigid part of the developed device was printed in polyethylene terephthalate glycol (PETG), while the electrodes were printed using a conductive filament PLA-CB composite. Details of the STL model sliced and printing conditions were selected based on previous works.^31^

### Conductive Filament Preconditioning

The electrodes (based on PLA-CB) placed inside the measurement platform were activated following a two-step protocol to increase the electroactive area and improve signals. First, a 0.5 M NaOH solution was placed inside the platform for PLA saponification in alkali media (chemical activation). Then, a second step of electrochemical activation was conducted using cyclic voltammetry (0.5 M NaOH, 0.1 V s^–1^ scan rate, from −1.0 V to +1.5 V, 10 cycles). After the activation process, the devices were thoroughly rinsed to eliminate the side product of filament degradation.

### Electroactive Area of the WE

The degree of PLA-CB filament activation was investigated by the inner-sphere redox 5 mM (in each component) [Fe­(CN)_6_]^4‑/3–^ probe in 0.1 M KNO_3_ under static conditions. CVs were carried out at 0.050 V s^–1^ from the −1 to +1 V potential window. The electroactive surface area of the WE after the activation treatment can be easily calculated through voltammetric studies probes by using Randles–Sevcik equation:

$$i_{p}=\left(2.69x10^{5}\right)\cdot n^{3/2}\cdot A\cdot D^{1/2}\cdot C\cdot v^{1/2}$$

where *i_p_* is the peak current (A), 2.69 × 10^5^ is a constant number (C mol^–1^ V^–1/2^); *n* is the number of electrons transferred in the redox reaction, D is the diffusion coefficient of the redox couple (cm^2^·s^–1^), *A* is the electroactive area of the electrode (cm^2^), *C* is the concentration of the redox molecule (mol·cm^–3^), and *v* the scan rate (in V·s^–1^). Using this method in the presence of [Fe­(CN)_6_]^4‑/3–^ as the redox probe, the electroactive area of our device was determined to be 0.0301 ± 0.004 cm^2^.

### Electronic Impedance Spectroscopy (EIS) Analysis

The fitting of the spectra using a Randle’s equivalent circuit allows us to determine the charge transfer resistance, R_CT_, of the different biotemplate-based micromotors. In the proposed circuit, the capacitor has been substituted by a constant phase element (CPE) to improve the fitting and account for deviations from ideal capacitive behavior (CPE = (*Q*(iω)^*n*^)^−1^, where *Q* and *n* are the magnitude and exponent parameters of the CPE). Thus, *R*_s_ is the electrolyte resistance, which is in series with the parallel combination of the CPE (that models the real behavior of a double layer as an imperfect capacitor) and the diffusional resistance element (*Z*_w_) of a faradaic reaction. Such an element is in series with *R*_CT_ as it is assumed that the rate of the faradaic reaction is controlled by the diffusion of the reactants to the electrode surface.

## Results and Discussion

### Synthesis, Characterization and Magnetic Motion of the Biotemplated Bacteria@Fe_3_O_4_@BiOCl Photocatalytic Micromotors

Figure 1 illustrates a schematic of the design of biotemplate-based multifunctional micromotors (A) and the proposed mechanism for bacterial growth inhibition (B). For a detailed protocol, please see the Experimental Section. Biotemplate approach allows a controlled and reproducible mold production with identical shape and size morphological features as well as chemical properties for biocompatible micromotors preparation. The diverse and well-defined natural morphologies of bacteria inherently facilitate the rational and reproducible design of material surface modifications. For example, *E. coli* is rod-shaped, while *S. aureus* is spherical. This allows for the fabrication of micromotors with reproducible shapes, which can be challenging to achieve through purely synthetic top-down or bottom-up methods. The different morphologies of these bacteria could result in an increased surface area for the controlled, well-ordered incorporation of magnetic and photocatalytic BiOCl nanoparticles, thus maximizing the guidance and photocatalytic abilities of the micromotors in bacterial inactivation. Second, using bacteria results in a cost-effective, potentially scalable procedure for obtaining large quantities of micromotors. Growing bacterial cultures is a relatively inexpensive and scalable way to produce many templates, especially compared to advanced lithographic techniques for microfabrication. In this work, *E. coli* or *S. aureus* bacteria were used as templates with different shape and size morphological features for modification with Fe_3_O_4_, resulting in a magnetic entity with full guidance and propulsion abilities. We followed previous template-based approaches with some modifications.^32–34^ For improved biocompatibility, we replaced paraformaldehyde with glutaraldehyde to perform the bacteria fixation prior to modification with the Fe_3_O_4_ nanoparticles.^35^ This is further supported by the scanning-electron microscopy (SEM) images in Figure 1C-a and C-d, where the characteristic morphology of *E. coli* and *S. aureus* bacteria remains intact after fixation. Next, the micromotors were modified with magnetic nanoparticles after overnight incubation. This decoration can be explained by the electrostatic interaction of the cationic Fe_3_O_4_ nanoparticles with the negative charge in the bacterial surface imparted by the glutaraldehyde and the phosphate groups of the outer bacteria membrane.^36,37^ The successful modification is reflected in the SEM of Figure 1C-b and C-e and in the energy-dispersive X-ray (EDX) images in Figure S1A. To obtain multifunctional micromotors with ROS generation abilities, BiOCl crystals were grown on the surface of the bacteria@Fe_3_O_4_ micromotors. To this end, a room-temperature hydrolysis method was adopted, using bismuth (III) nitrate pentahydrate as the bismuth source, NaCl as the chlorine source, and different solvents (acetic acid, HAc; ethylene glycol, EG; and glycerol, GL) to tailor crystal morphology by varying the viscosity of the solvent during the crystallization process. A flower-like morphology can be clearly observed, with defects and abundant areas for further ROS generation (see Figure 1C-c and C-f). For more details, see the Experimental Section. The effective element distribution is also clearly observed in the EDX mapping presented in Figure S1B.

**Figure 1:**
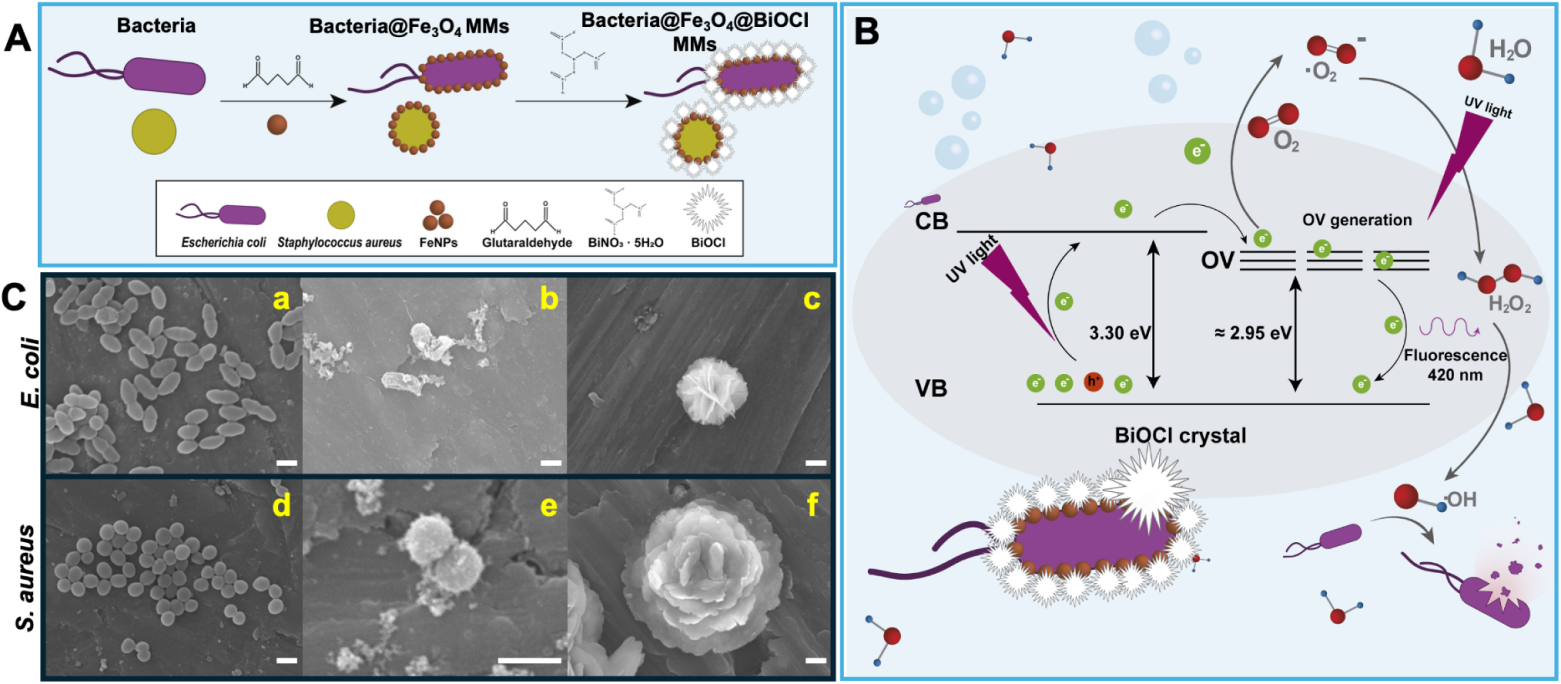
(A) Schematic of the synthesis of the magnetic biotemplated bacteria@Fe_3_O_4_@BiOCl photocatalytic micromotors and (B) mechanism schematics of ROS generation for bacterial growth inhibition. (C) SEM images of (a, d) glutaraldehyde-stabilized bacteria, (b, e) bacteria@Fe_3_O_4_ micromotors, and (c, f) bacteria@Fe_3_O_4_@BiOCl micromotors. Scale bars: 1 μm.

On the other hand, oxygen vacancies (OV) are critical for the development of photocatalytic micromotors because they play a key role in enhancing their reactivity and functionality.^38^ The generation of OV in our micromotors can be explained in terms of the OV generational regeneration. The radiation used in these experiments (375 nm) has enough energy to generate OV due to the low bond energy of Bi–O in the crystal structure. This radiation also promotes the transfer of electrons from the valence band to the conduction band. These electrons are then transferred to the OV, which is less energetic than the conduction band, with a longer emission wavelength (420 nm) (see Figure 1B for the proposed mechanism).

The difference in the energy between the OV and the valence band can be studied by a fluorescence emission spectrum by irradiating at 375 nm (see Figure S1C). These fluorescence emission spectra present their maximum peak around 420 nm, which corresponds to a calculated energy of 2.95 eV. The electrons present at OV are mainly responsible for ROS generation through two different mechanisms: dissociative and molecular channels. In the case of continuous irradiation, OV are not regenerated, and oxygen atoms resulting from the dissociation of molecular oxygen reoxidize the surface and block the OV, preventing the formation of new ROS.^39^ Attending to this mechanism, the interaction with light and the absorption capacity of BiOCl depend on its microstructure and morphology. As such, we studied the influence of the different solvents used (HAc, EG, and GL) on the morphology and absorption capacity of the micromotors. BiOCl formation can be explained via the generation of [Bi_2_O_2_]^2+^ intermediates. In this reaction, acidic aqueous conditions facilitate the instant reaction between Bi^3+^, water, and halide anions at room temperature, yielding the desired bismuth oxyhalide products.^40^ Also, the viscosity of the solvent used can affect the diffusion rate of the ions, regulating the crystal growth.^41^ Thus, as can be seen in the SEM images in Figure S2A different morphologies can be observed, with a clear trend in crystal size related to the viscosity of HAc (1.06 cP), EG (16.1 cP), and GL (934 cP). Flower-like crystals with a highly exposed area are observed when using HAc, while for the most viscous solvents, smaller crystals are observed. While other variables should be considered, the viscosity of the solvent plays a crucial role in the dispersion efficiency of the particles present in the solution. Because of this, the morphology, growth of the crystal structure, and photocatalytic properties are strongly affected by this characteristic.

To support the successful generation of the photocatalytic BiOCl nanoparticles and to get some insights into the synthetic mechanism, bacteria@Fe_3_O_4_@BiOCl micromotors using *E. coli* and *S. aureus* as biotemplates were analyzed by UV/vis absorption (see Figure S1D) and XPS analysis (see Figure S2B,C). The XPS survey spectrum reveals the presence of the main elements: Bi, O, and Cl. The individual XPS spectra are depicted in Figure S2C. In the case of Bi 4f, the peaks corresponding to Bi 4f_5/2_ and Bi 4f_7/2_ (typical of Bi^3+^ in BiOCl) were found at approximately 164.5 and 159.8  eV in all cases.^42,43^ A minor peak centered at 157.2  eV could be observed when using EG and GL for synthesis, which is less pronounced in HAc (see the area in the red square of Figure S2C). This peak can be attributed to Bi 4f in the zero valence state (Bi^0^).^42,43^ The observation of the O 1s spectrum reveals a peak at around 530.5 eV in all cases, representative of the Bi–O bonds in [Bi_2_O_2_]^2+^/BiOCl lattice.^42^ The Cl 2p spectrum was resolved into two peaks located at 200.0 and 198.5  eV, which correspond to the binding energies of Cl 2p_1/2_ and Cl 2p_3/2_, respectively.^44^ From the UV/vis spectra, in all cases, the characteristic peaks of the BiOCl crystals are present with the appearance of an adsorption peak at 375 nm. The micromotors prepared in EG and GL exhibit stronger UV and visible light absorption than those prepared in HAc, due mainly to the existence of metallic Bi^0^ nanoparticles as a consequence of the lower reaction yields caused by the higher viscosity of these solvents.^43^ Yet, the presence of Bi^0^ nanoparticles can cover the reaction sites on the surface of the BiOCl photocatalyst,^45^ resulting in lower ROS generation abilities, as will be further studied in photoelectrochemical studies. Please note that the baseline for *E. coli* and *S. aureus* micromotors prepared using HAc is lower than the baseline for all micromotors prepared using EG or GL. This fact further supports the presence of Bi^0^ nanoparticles and the lower reaction yields when using EG and GL.^43^ For further experiments, the micromotors prepared using HAc as the solvent were selected as optimal on account of their higher ability for ROS generation.

Then, micromotor magnetic motion was characterized toward the influence of applied voltage in the micromotor propulsion using a solenoid/rotating permanent magnet controlled by an Arduino board, as previously described by our research group.^46^ The magnitude of the applied magnetic field at each voltage is listed in Table S1. This magnitude was empirically determined by measuring the surface of the electromagnet using a teslameter. Micromotors maintain their magnetic propulsion abilities after modification with the BiOCl, as illustrated in the time-lapse images in Figure 2 and Video S1.

**Figure 2:**
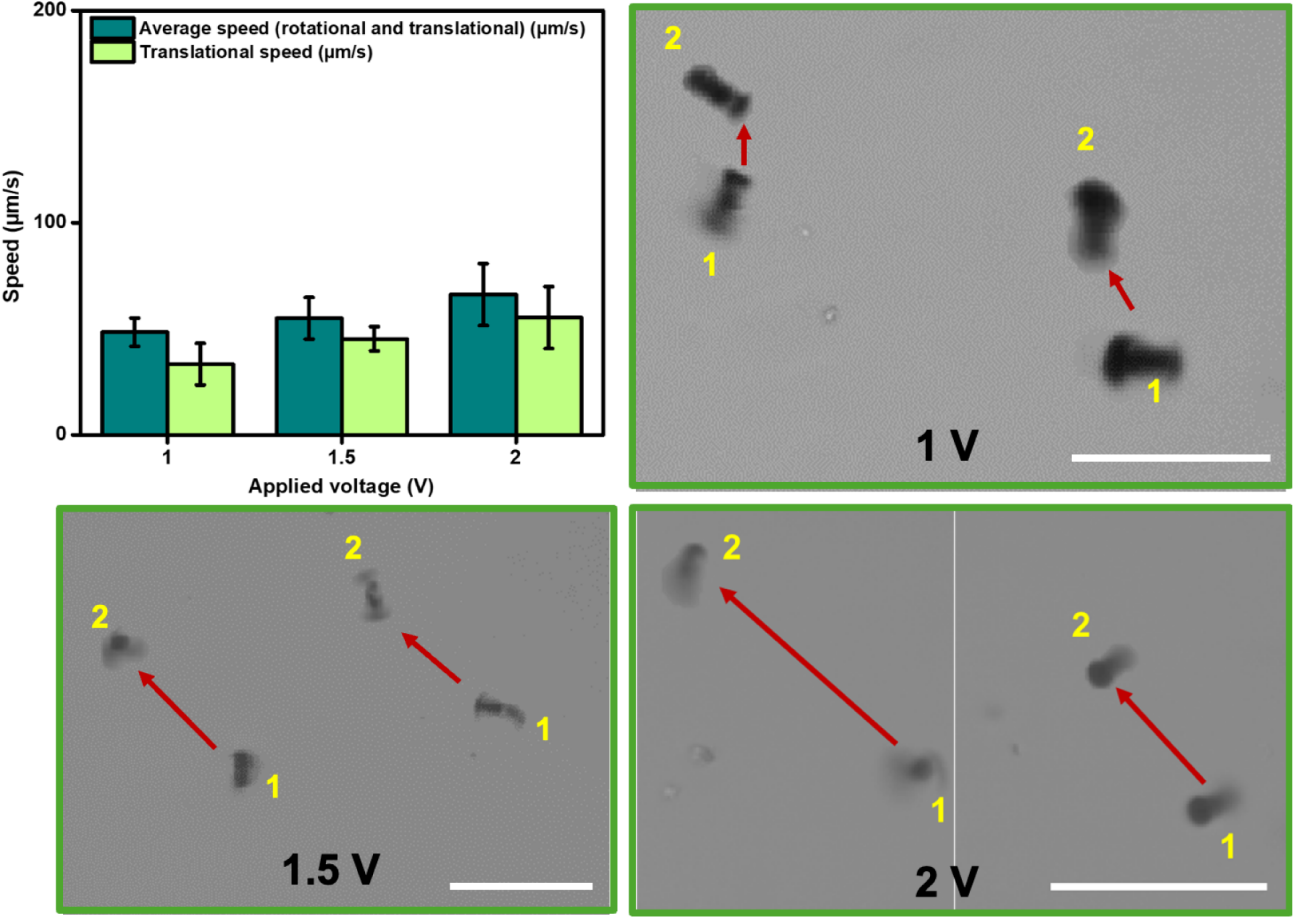
Magnetic propulsion of biotemplated bacteria@Fe_3_O_4_@BiOCl micromotors. Control of the speed upon the magnetic field (over a 1 s period, taken from Video S1) at each condition. Scale bars, 50 μm.

To study the propulsion, 1 μL of micromotor solution was placed on a glass slide on top of the inverted optical microscope. The tailored magnetic device was placed as a replacement for the microscope stage (for more details, see the Experimental Section). The desired voltage was applied, and videos were recorded at 20 frames per second, using both *E. coli* and *S. aureus* as biotemplates. Similar speeds with no statistical differences were obtained. For simplicity, the data in Figure 2 corresponds to bacteria@Fe_3_O_4_@BiOCl­(HAc) micromotors using *E. coli* as the biotemplate. The average speed was calculated using the dedicated software on the microscopy. We calculated the translational speed (or specific displacement). To measure the micromotor translational speed, a coordinate where the micromotor is located at time 0 was used as reference “A” and a coordinate after the movement was used as reference “B.” As such, it was possible to calculate the displacement produced between points A and B and divide it by the exact time taken to travel from point A to point B, thus obtaining an exact translational speed. To calculate the translational speed, the displacement (Δx) was calculated as Euclidean distance **(Δx)** ^**2**^ **=**a^2^+b^2^, where “a” corresponds to the coordinates at point “a” (the point where the micromotor is located before starting movement), “b” corresponds to the coordinates at point “b” (the point where the micromotor is located at the final of the move), and (Δx) is the displacement produced by the micromotor between the references “a” and “b”. As can be seen in Figure 2, the micromotor speed increases along with the voltage, revealing the ability to control motion and propulsion. Rumble and tumble motion during the movement is observed, with specific displacement from points 1 to 2. With all applied voltages, no significant differences in the speeds are observed.

### Characterization and Assessment of the Photocatalytic Properties of Bacteria@Fe_3_O_4_@BiOCl Micromotors

The ability of micromotors for ROS generation and monitorization was studied using a tailored 3D-printed (photo)­electrochemical device with a dedicated chamber to hold the micromotor solution, as depicted in Figure 3A. For more details, please refer to the Experimental Section. We designed a device equipped with an LED source controlled by an Arduino board to apply UV radiation within the desired range, a black box, and a platform to accommodate the 3D-printed electrochemical cell, which is connected to the potentiostat. In this way, the micromotors can be placed on the 3D-printed electrodes inside the black box, which is closed to allow light ON-OFF irradiation cycles (micromotors activation ON state and deactivation OFF state) on demand and (photo)­electrochemical monitoring.

**Figure 3:**
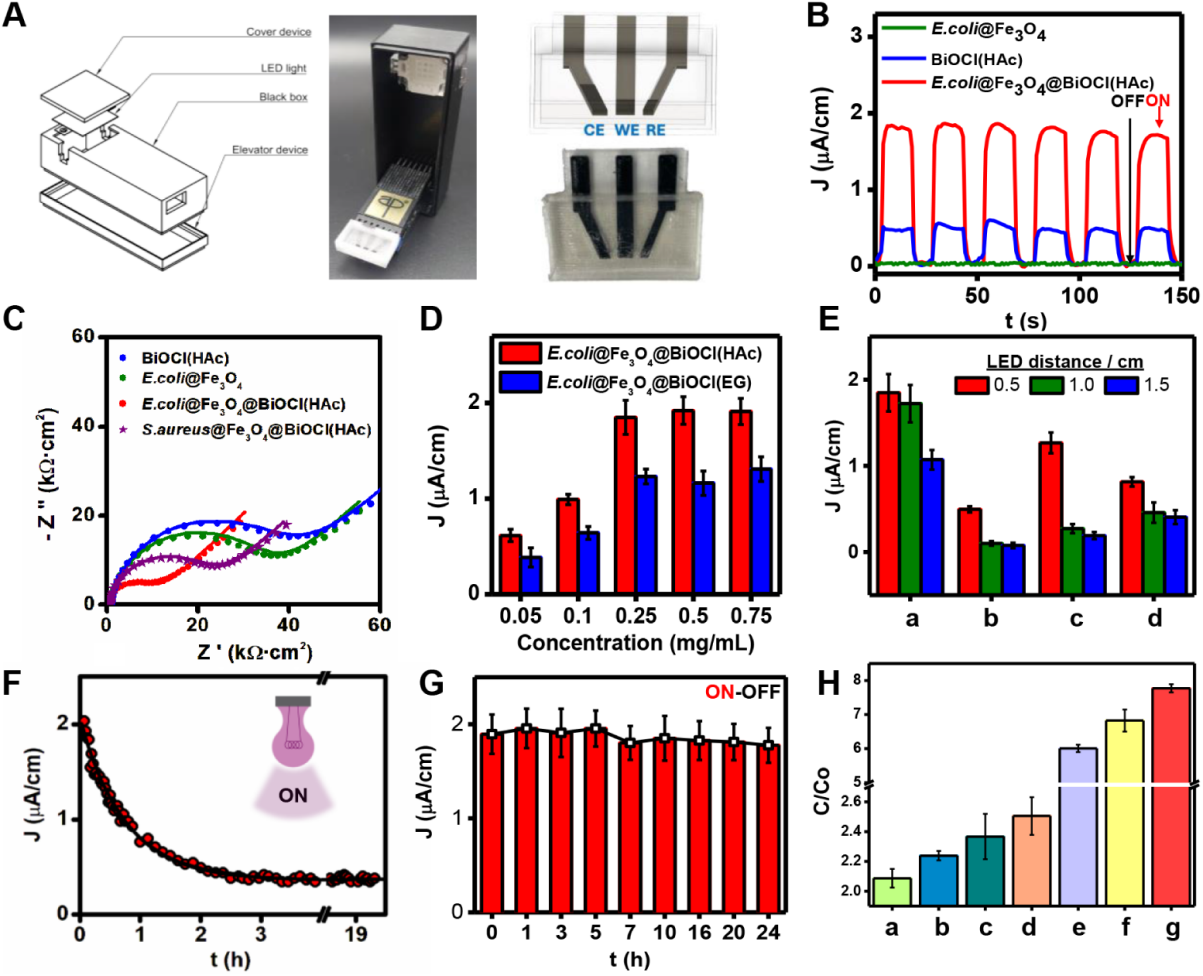
3D-printed device for the study of the photoelectrochemical behavior of the magnetic bacteria@Fe_3_O_4_@BiOCl micromotors for bacterial inactivation. (A) Schematic and corresponding picture of the device containing the LED light and the 3D-printed microchip with a tailored reservoir to accommodate the micromotors, which is shown in detail in the right part. CE: Counter electrode; WE: working electrode; RE: reference electrode. (B) Photocurrent response (intermittent radiation on–off cycles) for BiOCl and *E. coli*@Fe_3_O_4_@BiOCl­(HAc) micromotors in PBS 0.1 M (conditions, 0.5 cm LED height, 375 nm, 10 W, Eap 1.0 V, 15s ON-OFF irradiation cycles). (C) EIS Nyquist plots of the different stages of the micromotors synthesis. (D) Evaluation of micromotor concentration and (E) electrode-LED distance on the current density measurement for a) *E. coli*@Fe_3_O_4_@BiOCl­(HAc), b) *S.aureus*@Fe_3_O_4_@BiOCl­(HAc), c) *E. coli*@Fe_3_O_4_@BiOCl­(EG), d) *S.aureus*@Fe_3_O_4_@BiOCl­(EG) (F) Monitoring of the photocurrent response irradiating continuously (conditions, 0.5 cm LED height, 375 nm, 10 W, Eap 1.0 V) and (G) evaluation of the photodurability of the *E. coli*@Fe_3_O_4_@BiOCl­(HAc) micromotors (conditions, 0.5 cm LED height, 10 W, Eap 1.0 V, 15 s ON-OFF irradiation cycles). (H) Inhibition of *E. coli* growth at 7 h for a) intermittent UV light bacterium and micromotors, b) intermittent UV light bacterium and static micromotors, c) continuous UV light bacterium and micromotors, d) darkness bacteria and micromotors, e) continuous UV light bacterium, f) intermittent UV light bacterium, and g) darkness bacteria .

First, we characterized the tailored 3D-printed electrochemical cell. The device was printed using PETg as an insulating material due to its good flexibility, chemical resistance, easily processed, and well-sterilized properties.^47^ A PETg-CB filament was used to print the three electrodes, and their electrochemical performance was improved using an activation protocol. The electrode surface was first characterized by SEM. As shown in Figure S3A­(a and b), clear differences can be observed between the surface of the as-printed electrodes, and once they have been activated. Additionally, EDX analysis (Figure S3B) demonstrates that the selected activation process allows for the overexposure of carbon particles on the surface of the electrodes, which further explains the differences in the electrochemical performance before and after the activation protocol. This overexposure is evidenced by EDX through the comparison of the elemental ratios: upon activation, a significant amount of carbon becomes exposed at the surface. Although additional oxygen-containing groups are also generated during the process, the amount of carbon detected on the activated surfaces is consistently higher compared to the nonactivated ones. However, it is important to note that in Figure S3B, we are comparing the relative presence of C and O for each type of surface, but the total amount of exposed material is substantially greater in the activated devices than in the as-printed ones.

Since this device must also be able to host the biotemplate for long periods without deteriorating, its stability after sterilization was studied. Thus, the electrochemical cell was first sterilized using a disinfecting technique employing UV light. Then, the devices were filled with 0.1 M PBS solution for 24 h. No leakage was observed, indicating that the mechanical properties of the device’s PETg cover are not appreciably deteriorated after this process. To check that electrochemical performance is preserved after the disinfection process, cyclic voltammograms in the presence of [Fe­(CN)_6_]^3–/4–^ inner-sphere redox probe (0.1 M KNO_3_) were taken before and after the sterilization process (see Figure S2C). These measurements provide evidence that the maximum current intensities are maintained, as well as the *E*_1/2_ and ΔE values. After the sterilization protocol, we found *E*_1/2_ and ΔE good values of 77 ± 2 and 118 ± 5 mV, respectively. Another important factor, as it is the degree of PLA-CB filament activation, is estimated using the same experimental conditions and varying the scan rate to record several cyclic voltammograms in a fixed (−0.5 to +0.7 V) potential window (Figure S3D). Such activation defines the morphology of the materials that constitute the WE surface and defined its electroactive area (0.030 ± 0.004 cm^2^) which was calculated through voltametric studies of standard redox probes by employing the Randles-Sevcik equation^48,49^ (details are presented in Figures S3E, and S4).

After the characterization of the 3D electrodes, we characterized the photocatalytic properties of the biotemplated bacteria@Fe_3_O_4_@BiOCl micromotors. Photocurrent–time responses were recorded to gain more insights into the interfacial charge transfer dynamics of the *E. coli*@Fe_3_O_4_@BiOCl­(HAc) micromotors. Control experiments using BiOCl and the magnetic *E. coli*@Fe_3_O_4_ micromotors were also included. The as-obtained responses in Figure 3B can be directly correlated with the recombination efficiency of the photogenerated carriers. We carried out several ON-OFF irradiation cycles to record the transient photocurrent ROS responses. In light with the mechanism stated before, the semiconductor produces holes in the VB when exposed to light since the electrons are promoted to the CB. It is generally accepted that the effectiveness of electron separation concerning holes is mandatory to achieve a successful photocatalytic process. In particular, greater photocatalytic activity would result from longer-living photogenerated electrons and holes, which would be implied by a greater photocurrent.^50^ The I-t curves for BiOCl and *E. coli*@Fe_3_O_4_@BiOCl­(HAc) micromotors reflect the high photocatalytic activity, with a sharp increase in the current after light irradiation, swiftly reaching a steady state with a high precision (RSD < 2%, *n* = 9). When the light is turned off, the current swiftly recovers to its dark-current state. The intensity was dramatically higher when the micromotors were used in comparison with the BiOCl due to the heterojunction photocatalysts formed by the combination of Fe_3_O_4_ and BiOCl, in comparison with the BiOCl semiconductor.^51^ No photocatalytic activity, as expected, was noted in *E. coli*@Fe_3_O_4_ micromotors.

Interestingly, as can be seen in the EIS Nyquist plots of Figure 3C the arc of the diameter is significantly smaller for both biotemplate @Fe_3_O_4_@BiOCl (HAc) micromotors as compared with BiOCl­(HAc), suggesting a faster interfacial charge transfer. The R_CT_ values that support this trend are summarized in Table S2. Indeed, biotemplated-based micromotors present smaller R_CT_ (kΩ·cm^2^) values (16 ± 2 and 28 ± 2 for *E. coli* and *S. aureus*, respectively) than those observed for the free BiOCl (52 ± 2) and *E. coli*@Fe_3_O_4_ (41 ± 3), indicating that the interfacial charge-transfer process occurred quickly in the *bacteria*@Fe_3_O_4_@BiOCl micromotors. The same trend was observed when EG was employed as the crystallization solvent. Interestingly, this faster interfacial charge transfer is in agreement with the higher photocatalytic activity of the *bacteria*@Fe_3_O_4_@BiOCl micromotors in comparison with BiOCl, which is also in agreement with the literature in light with the fact that the longer living photogenerated electrons and holes are generated, increasing the photocatalytic activity.^50,52^ Such results indicate the optimal photocatalytic activity of the bacteria-based micromotors and their ability to generate a swarming of ROS.

For further bacterial growth inhibition, the effects of micromotor concentration, solvent, and biotemplate employed during the crystallization process (Figure 3D) and the distance of the LED to the WE surface, in addition to power (Figure 3E), were investigated. The current intensity increases with the concentration of biotemplated micromotors up to a concentration of 0.25 mg/mL and remained constant. As such, this concentration was selected as optimal. Regarding the LED power and position, 0.5 cm is the minimum distance at which the light can be placed relative to the WE, based on both the design of the measurement platform and the volume of the sample solution. As expected, the intensity decreases as the LED source is moved away from the WE, although there is no linear relationship with it. A similar conclusion was obtained after the evaluation of the LEDs’ power (1, 5, and 10 W). The lower-power LED provides intensity signals that, in some samples, are not distinguishable from noise. The signal intensity is sufficient when 5 W LED is employed; however, the interdevice response is poorly reproducible. For 10 W, a high interdevice reproducibility is achieved (RSD < 4, *n* = 15), with a good device stability, in addition to a good intensity, and was then selected as optimal. Regarding the crystallization conditions and attending to the electrochemical characterization *E. coli* micromotors present better performance (R_CT_ = 16 ± 2 kΩ·cm^2^) than *S. aureus* micromotors (R_CT_ = 28 ± 2 kΩ·cm^2^), as well as using HAc as the solvent was shown to be a better option than EG (see Table S2). These results are coherent with the crystallinity observed by SEM (Figure S1). According to these results, *E. coli*@Fe_3_O_4_@BiOCl (HAc) micromotors were selected to carry out the inhibition growth studies.

The response of the *E. coli*@Fe_3_O_4_@BiOCl (HAc) was continuously monitored for ca. 24 h, without stopping irradiation once it had been started (Figure 3F, constant ON state). Analyzing the response obtained, it is observed that it decreases exponentially with time. In the first hour, approximately 60% of the initial signal is lost, and the stationary response is reached when the system has been irradiated for around 1.5 h, being the average density current only 20% of the initial one, probably due to the saturation of the OV when they are not regenerated under light with the proposed mechanism (Figure 1B). We also monitored the response under the optimal conditions (0.5 cm LED height, 10 W, Eap 1.0 V) by using cycles of 15 s of irradiation (ON) and 10 s of darkness (OFF) (total time 24 h) to allow the regeneration of the OV on the photocatalyst surface. During the time interval between one measurement and another, both the electrochemical device and the sample solution were kept in total darkness (Figure 3G, OFF-ON cycles). Interestingly, until hour 5, we observed that the measured intensity remains constant (2.0 ± 0.2 μA·cm^–2^) while after that time, only a slight decrease (less than 10%, 1.8 ± 0.2 μA·cm^–2^) was observed. These results allow us to state that the designed *E. coli*@Fe_3_O_4_@BiOCl­(HAc) maintains its properties for at least 24 h of applying ON-OFF cycles of radiation. This excellent photostability allows us to ensure the controlled ROS production required for bacterial inactivation processes.^53,54^

On the other hand, the studies also aimed to evaluate the inhibition of *E. coli* growth under different conditions, as shown in Figure 3H. The experiment was carried out using turbidimetry (OD600) measurements over a period of 7 h, with one measurement taken per hour. The study involved continuous UV irradiation and intermittent UV irradiation, with the results being consistent with the previous photocatalytic capabilities of *E. coli*@Fe_3_O_4_@BiOCl micromotors. Control experiments were also performed in the dark, in static mode, and using bacterial cultures without micromotors.

The first measurement was taken without any previous time culture and was considered the initial concentration (Co). The subsequent measurements were evaluated in comparison to the initial concentration (C/Co) to determine the bacterial growth under UV light assayed and optimal conditions (an LED height of 0.5 cm and 10 W power). The lowest bacterial inhibition (higher bacterial growth, highest C/Co ratio) was observed after 7 h of culture of bacteria in the dark (bacteria control), and the highest bacterial inhibition growth (lowest bacterial growth, lowest C/Co ratio) using moving *E. coli*@Fe_3_O_4_@BiOCl­(HAc) micromotors under intermittent light as optimum conditions. This indicates that the inhibition of bacteria is caused by the photocatalytic effect of BiOCl present in the micromotor surface due to ROS generation under intermittent irradiation. Static micromotor control under intermittent irradiation was also performed, showing less efficiency in comparison with the magnetic mode and demonstrating the dynamic action of the magnetic and photocatalytic micromotors acting as efficient microswimmers. In addition, bacterial cultures without micromotors, irradiated by continuous UV light and intermittent UV light, showed a similar trend, with a slightly lower concentration of bacteria at the end of the experiment compared to experiments conducted in the dark. It is important to note that the UV light used in these experiments is in the UV-A region (375 nm), which is not commonly used for disinfection unless DNA damage is generated, thereby reducing the normal growth rate. Furthermore, the results indicate that the inhibition in the normal growth rate of the bacterial culture in contact with irradiated micromotors is more pronounced using intermittent UV irradiation than with continuous UV irradiation, with almost 100% inhibition of bacterial growth.

## Conclusions

In this work, magnetic photocatalytic micromotors based on *E. coli* or *S. aureus* as biotemplates have been successfully designed and developed. It has been shown that multifunctional micromotors designed to incorporate both magnetic and photocatalytic functional materials for controlled guidance and ROS generation have proven to be effective in inhibiting bacterial growth. The high photostability of these micromotors is also compatible with long culture times, demonstrating their enormous potential for studying and controlling bacterial growth inhibition. Given the relevance of the global problem of bacterial resistance, this work represents a further step in demonstrating the enormous potential of micromotor technology for this purpose, where micromotor design and rigorous analytical control through the incorporation of appropriate detection systems are essential to further address this challenge. Current and future efforts of our laboratory are aimed at the study and reliable monitoring of the bacterial inactivation process with disruptive technologies at the nano- and microscale.

## Supplementary Material





## Notes

Figures S1 (Biotemplated micromotor characterization); S2 (SEM images and XRD characterization of the micromotors); S3 (SEM images and electrode characterization); S4 (equivalent Randless circuit); Table S1 (Relation between the applied voltage and the magnetic field applied) and S2 (R_CT_ values obtained from the fitting of EIS experimental using Nyquist equivalent circuit) (PDF)
Magnetic motion of the micromotors (MP4)

## References

[1] Chambers H. F., DeLeo F. R. (2009). Waves of Resistance: Staphylococcus aureus in the Antibiotic
era. Nat. Rev. Microbiol..

[2] Murray C. J. L., Ikuta K. S., Sharara F., Swetschinski L., Robles Aguilar G., Gray A., Han C., Bisignano C., Rao P., Wool E. (2022). Global
Burden of Bacterial Antimicrobial Resistance
in 2019: A Systematic Analysis. Lancet.

[3] Paxton W. F., Kistler K. C., Olmeda C. C., Sen A., St S. K., Cao Y., Mallouk T. E., Lammert P. E., Crespi V. H., Crespi V. H. (2004). Catalytic
Nanomotors: Autonomous Movement of Striped Nanorods. J. Am. Chem. Soc..

[4] Ozin G. A., Manners I., Fournier-Bidoz S., Arsenault A. (2005). Dream Nanomachines. Adv. Mater..

[5] Ghosh A., Fischer P. (2009). Controlled Propulsion
of Artificial Magnetic Nanostructured
Propellers. Nano Lett..

[6] Gao W., Sattayasamitsathit S., Orozco J., Wang J. (2011). Highly Efficient Catalytic
Microengines: Template Electrosynthesis of Polyaniline/Platinum Microtubes. J. Am. Chem. Soc..

[7] Mei Y., Solovev A. A., Sanchez S., Schmidt O. G. (2011). Rolled-up Nanotech
on Polymers: From Basic Perception to Self-Propelled Catalytic Microengines. Chem. Soc. Rev..

[8] Garcia-Gradilla V., Orozco J., Sattayasamitsathit S., Soto F., Kuralay F., Pourazary A., Katzenberg A., Gao W., Shen Y., Wang J. (2013). Functionalized
Ultrasound-Propelled Magnetically Guided Nanomotors:
Toward Practical Biomedical Applications. ACS
Nano.

[9] Xu L., Mou F., Gong H., Luo M., Guan J. (2017). Light-Driven Micro/Nanomotors:
From Fundamentals to Applications. Chem. Soc.
Rev..

[10] Wang W., Duan W., Ahmed S., Sen A., Mallouk T. E. (2015). From One
to Many: Dynamic Assembly and Collective Behavior of Self-Propelled
Colloidal Motors. Acc. Chem. Res..

[11] Singh V. V., Jurado-Sánchez B., Sattayasamitsathit S., Orozco J., Li J., Galarnyk M., Fedorak Y., Wang J. (2015). Multifunctional Silver-Exchanged Zeolite Micromotors for Catalytic
Detoxification of Chemical and Biological Threats. Adv. Funct. Mater..

[12] Li J., Singh V. V., Sattayasamitsathit S., Orozco J., Kaufmann K., Dong R., Gao W., Jurado-Sanchez B., Fedorak Y., Wang J. (2014). Water-Driven Micromotors
for Rapid
Photocatalytic Degradation of Biological and Chemical Warfare Agents. ACS Nano.

[13] Mushtaq F., Guerrero M., Sakar M. S., Hoop M., Lindo A. M., Sort J., Chen X., Nelson B. J., Pellicer E., Pané S. (2015). Magnetically
driven Bi_2_O_3_/BiOCl-based
hybrid microrobots for photocatalytic water remediation. J. Mater. Chem. A.

[14] Xie L., Pang X., Yan X., Dai Q., Lin H., Ye J., Cheng Y., Zhao Q., Ma X., Zhang X. (2020). Photoacoustic Imaging-Trackable Magnetic Microswimmers
for Pathogenic
Bacterial Infection Treatment. ACS Nano.

[15] Stanton M. M., Park B.-W., Vilela D., Bente K., Faivre D., Sitti M., Sánchez S. (2017). Magnetotactic Bacteria Powered Biohybrids
Target *E. Coli* Biofilms. ACS Nano.

[16] Gao W., Feng X., Pei A., Kane C. R., Tam R., Hennessy C., Wang J. (2014). Bioinspired
Helical Microswimmers
Based on Vascular Plants. Nano Lett..

[17] Mayorga-Martinez C. C., Fojtů M., Vyskočil J., Cho N.-J., Pumera M. (2022). Pollen-Based
Magnetic Microrobots are Mediated by Electrostatic Forces to Attract,
Manipulate, and Kill Cancer Cells. Adv. Funct.
Mater..

[18] Zhang F., Li Z., Chen C., Luan H., Fang R. H., Zhang L., Wang J. (2024). Biohybrid Microalgae Robots: Design, Fabrication, Materials, and
Applications. Adv. Mater..

[19] Li Z., Liu T., Sun X., Zhou Q., Yan X. (2024). Natural Algae-Inspired
Microrobots for Emerging Biomedical Applications and Beyond. Cell Rep. Phys. Sci..

[20] Wu Z., Li T., Li J., Gao W., Xu T., Christianson C., Gao W., Galarnyk M., He Q., Zhang L. (2014). Turning
Erythrocytes into Functional Micromotors. ACS
Nano.

[21] Wu Z., esteban-Fernández de Ávila B., Martín A., Christianson C., Gao W., Thamphiwatana S. K., Escarpa A., He Q., Zhang L., Wang J. (2015). RBC Micromotors
Carrying Multiple Cargos Towards Potential Theranostic Applications. Nanoscale.

[22] Medina-Sánchez M., Schwarz L., Meyer A. K., Hebenstreit F., Schmidt O. G. (2016). Cellular Cargo Delivery:
Toward Assisted Fertilization
by Sperm-Carrying Micromotors. Nano Lett..

[23] Magdanz V., Sanchez S., Schmidt O. G. (2013). Development of a Sperm-Flagella Driven
Micro-Bio-Robot. Adv. Mater..

[24] Xu H., Medina-Sánchez M., Magdanz V., Schwarz L., Hebenstreit F., Schmidt O. G. (2018). Sperm-Hybrid Micromotor for Targeted
Drug Delivery. ACS Nano.

[25] Chen Q., Tang S., Li Y., Cong Z., Lu D., Yang Q., Zhang X., Wu S. (2021). Multifunctional Metal–Organic
Framework Exoskeletons Protect Biohybrid Sperm Microrobots for Active
Drug Delivery from the Surrounding Threats. ACS Appl. Mater. Interfaces.

[26] Buss N., Yasa O., Alapan Y., Akolpoglu M. B., Sitti M. (2020). Nanoerythrosome-Functionalized Biohybrid
Microswimmers. APL Bioeng..

[27] Kabandana G. K. M., Jones C. G., Sharifi S. K., Chen C. (2020). 3D-Printed Microfluidic
Devices for Enhanced Online Sampling and Direct Optical Measurements. ACS Sens..

[28] Kharat, V. J. ; Singh, P. ; Raju, G. S. ; Yadav, D. K. ; Gupta, M. S. ; Arun, V. ; Majeed, A. H. ; Singh, N. Additive Manufacturing (3D Printing): A Review of Materials, Methods, Applications and Challenges. Mater. Tod. Proc., 2023, 10.1016/j.matpr.2023.11.033.

[29] McGlennen M., Dieser M., Foreman C. M., Warnat S. (2023). Using Electrochemical
Impedance Spectroscopy to Study Biofilm Growth in A 3D-Printed Flow
Cell System. Biosens. Bioelectron. :X.

[30] Dhall A., Ramjee R., Oh M. J., Tao K., Hwang G. (2022). A 3D-Printed
Customizable Platform for Multiplex Dynamic Biofilm Studies. Adv. Mater. Technol..

[31] Hernández-Rodríguez J. F., Rojas D., Escarpa A. (2023). Electrochemical Fluidic Fused Filament
Fabricated Devices (eF4D): In-Channel Electrode Activation. Sens. Actuat. B: chem..

[32] de
la Asunción-Nadal V., Franco C., Veciana A., Ning S., Terzopoulou A., Sevim S., Chen X.-Z., Gong D., Cai J., Wendel-Garcia P. D. (2022). MoSBOTs: Magnetically Driven Biotemplated MoS_2_-Based Microrobots
for Biomedical Applications. Small.

[33] Terzopoulou A., Palacios-Corella M., Franco C., Sevim S., Dysli T., Mushtaq F., Romero-Angel M., Martí-Gastaldo C., Gong D., Cai J. (2022). Biotemplating of Metal–Organic
Framework Nanocrystals for Applications in Small-Scale Robotics. Adv. Funct. Mater..

[34] Yan X., Zhou Q., Yu J., Xu T., Deng Y., Tang T., Feng Q., Bian L., Zhang Y., Ferreira A. (2015). Magnetite
Nanostructured Porous Hollow Helical
Microswimmers for Targeted Delivery. Adv. Funct.
Mater..

[35] Gong D., Cai J., Celi N., Feng L., Jiang Y., Zhang D. (2018). Bio-Inspired
Magnetic Helical Microswimmers Made of Nickel-Plated Spirulina with
Enhanced Propulsion Velocity. J. Magn. Magn.
Mater..

[36] Weiss L., Zeigel R., Jung O. S., Bross I. D. J. (1972). Binding of Positively
Charged Particles to Glutaraldehyde-Fixed Human Erythrocytes. Exp. Cell Res..

[37] Tayade N. T., Arjunwadkar P. R. (2017). Magnetic
Behavior of Fe_3_O_4_ Nanoparticles
on Post-HCL Treatment in Synthesis. Int. J.
Pure Appl. Phys..

[38] Wang Q., Dong R., Yang Q., Wang J., Xu S., Cai Y. (2020). Highly Efficient Visible-Light-Driven Oxygen-Vacancy-Based Cu_2+1_O Micromotors with Biocompatible Fuels. Nanoscale Horiz..

[39] Huang Y., Li H., Balogun M.-S., Liu W., Tong Y., Lu X., Ji H. (2014). Oxygen Vacancy Induced Bismuth Oxyiodide with Remarkably Increased
Visible-Light Absorption and Superior Photocatalytic Performance. ACS Appl. Mater. Interfaces.

[40] Gnayem H., Sasson Y. (2013). Hierarchical Nanostructured
3D Flowerlike BiOClxBr1–x
Semiconductors with Exceptional Visible Light Photocatalytic Activity. ACS Catal..

[41] Hu J., Weng S., Zheng Z., Pei Z., Huang M., Liu P. (2014). Solvents Mediated-Synthesis of BiOI Photocatalysts with Tunable Morphologies
and Their Visible-Light Driven Photocatalytic Performances in Removing
of Arsenic from Water. J. Hazard. Mater..

[42] Yang J., Liang Y., Li K., Yang G., Zhu Y., Liu S., Lei W. (2018). New Reaction
Pathway Induced by the Synergistic Effects
of Bi Plasmon and La^3+^ Doping for Efficient visible Light
Photocatalytic Reaction on BiOCl. Appl. Surf.
Sci..

[43] Liu H., Huang J., Chen J., Zhong J., Li J., Ma D. (2020). Influence
of Different Solvents on the Preparation and Photocatalytic
Property of BiOCl Toward Decontamination of Phenol and Perfluorooctanoic
Acid. Chem. Phys. Lett..

[44] Xu L., Yan P., Li H., Ling S., Xia J., Qiu J., Xu Q., Li H., Yuan S. (2017). Metallic Bi Self-Doping
BiOCl Composites:
Synthesis and Enhanced Photoelectrochemical Performance. Mater. Lett..

[45] Zhu G., Hojamberdiev M., Zhang S., Din S. T. U., Yang W. (2019). Enhancing
Visible-Light-Induced Photocatalytic Activity of BiOI Microspheres
for NO Removal by Synchronous Coupling with Bi Metal and Graphene. Appl. Surf. Sci..

[46] Cuntín-Abal C., Bujalance-Fernández J., Yuan K., Arribi A., Jurado-Sánchez B., Escarpa A. (2024). Magnetic Bacteriophage-Engineered
Janus Micromotors for Selective Bacteria Capture and Detection. Adv. Funct. Mater..

[47] Szykiedans K., Credo W., Osiński D. (2017). Selected Mechanical Properties of
PETG 3-D Prints. Proc. Eng..

[48] Lavagnini I., Antiochia R., Magno F. (2004). An Extended Method for the Practical
Evaluation of the Standard Rate Constant from Cyclic Voltammetric
Data. Electroanalysis.

[49] Sánchez S., Pumera M., Cabruja E., Fàbregas E. (2007). Carbon Nanotube/Polysulfone
Composite Screen-Printed Electrochemical Enzyme Biosensors. Analyst.

[50] Xiang Q., Yu J., Jaroniec M. (2011). Preparation and Enhanced Visible-Light Photocatalytic
H_2_-Production Activity of Graphene/C3N4 Composites. J. Phys. Chem. C.

[51] Li Y., Jiang H., Wang X., Hong X., Liang B. (2021). Recent Advances
in Bismuth Oxyhalide Photocatalysts for Degradation of Organic Pollutants
in Wastewater. RSC Adv..

[52] Xia J., Di J., Yin S., Xu H., Zhang J., Xu Y., Xu L., Li H., Ji M. (2014). Facile Fabrication of the Visible-Light-Driven
Bi_2_WO_6_/BiOBr Composite with Enhanced Photocatalytic
Activity. RSC Adv..

[53] Kacena M. A., Merrell G. A., Manfredi B., Smith E. E., Klaus D. M., Todd P. (1999). Bacterial Growth in
Space Flight: Logistic Growth Curve Parameters
for *Escherichia coli* and Bacillus subtilis. Appl. Microbiol. Biotechnol..

[54] Lindqvist R. (2006). Estimation
of Staphylococcus aureus Growth Parameters from Turbidity Data: Characterization
of Strain Variation and Comparison of Methods. Appl. Environ. Microbiol..

