# Supplementary material for: Biotemplated Photocatalytic Micromotors for Effective Inhibition of Bacterial Growth
Source: Chem Mater. 2025 Aug 19;37(17):6512–21. doi: 10.1021/acs.chemmater.5c00885 (PMC12424522; doi:10.1021/acs.chemmater.5c00885)
Supplement: Supplementary file 1 [file cm5c00885_si_001.pdf]

Supporting Information

Biotemplated photocatalytic micromotors for effective inhibition of bacterial growth

Carmen Cuntín-Abal,[a] Miriam Chávez,[a] Beatriz Jurado-Sánchez,[a,b] and Alberto Escarpa*[a,b]*

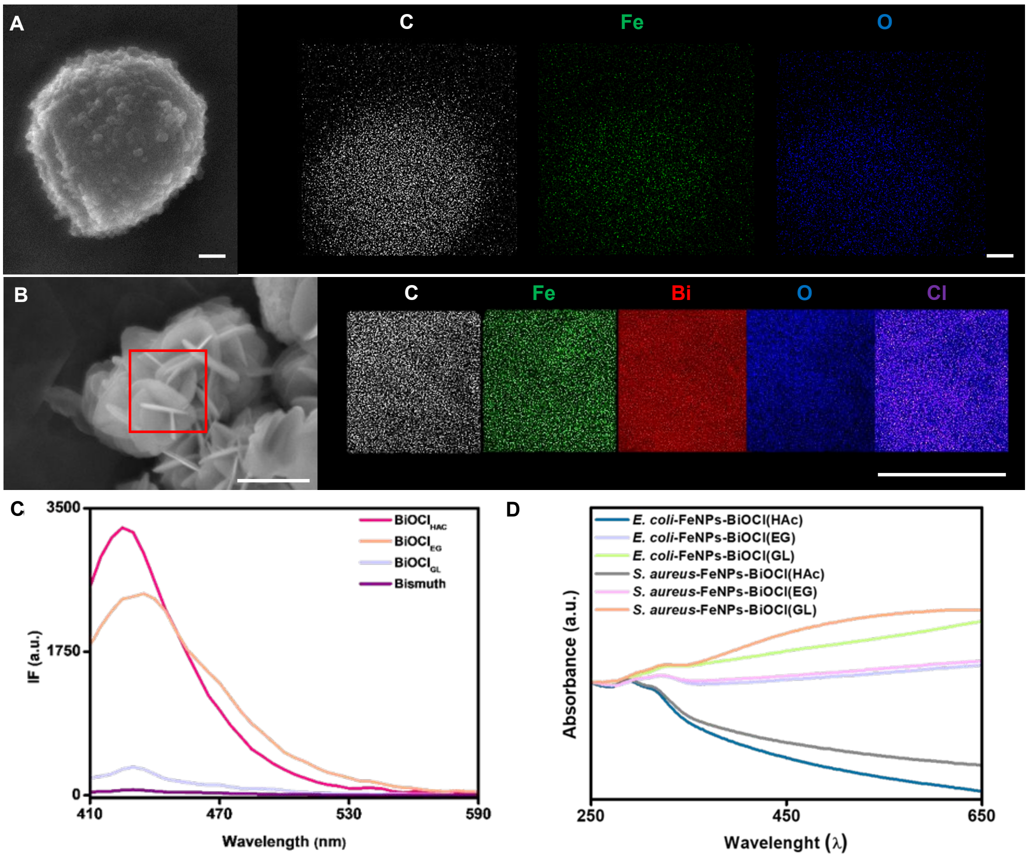

Figure S1. Biotemplated micromotor characterization. (A) SEM and corresponding EDX mapping of a bacteria@Fe_3O_4 and (B) a bacteria@Fe_3O_4 @BiOCl micromotor. Scale bars, 100 nm. (C) Fluorescence emission spectrum ($\lambda_{xcitation}$=375 nm) of the different BiOCl crystallizations and (D) Corresponding UV/VIS spectra of the micromotors synthetized in different conditions.

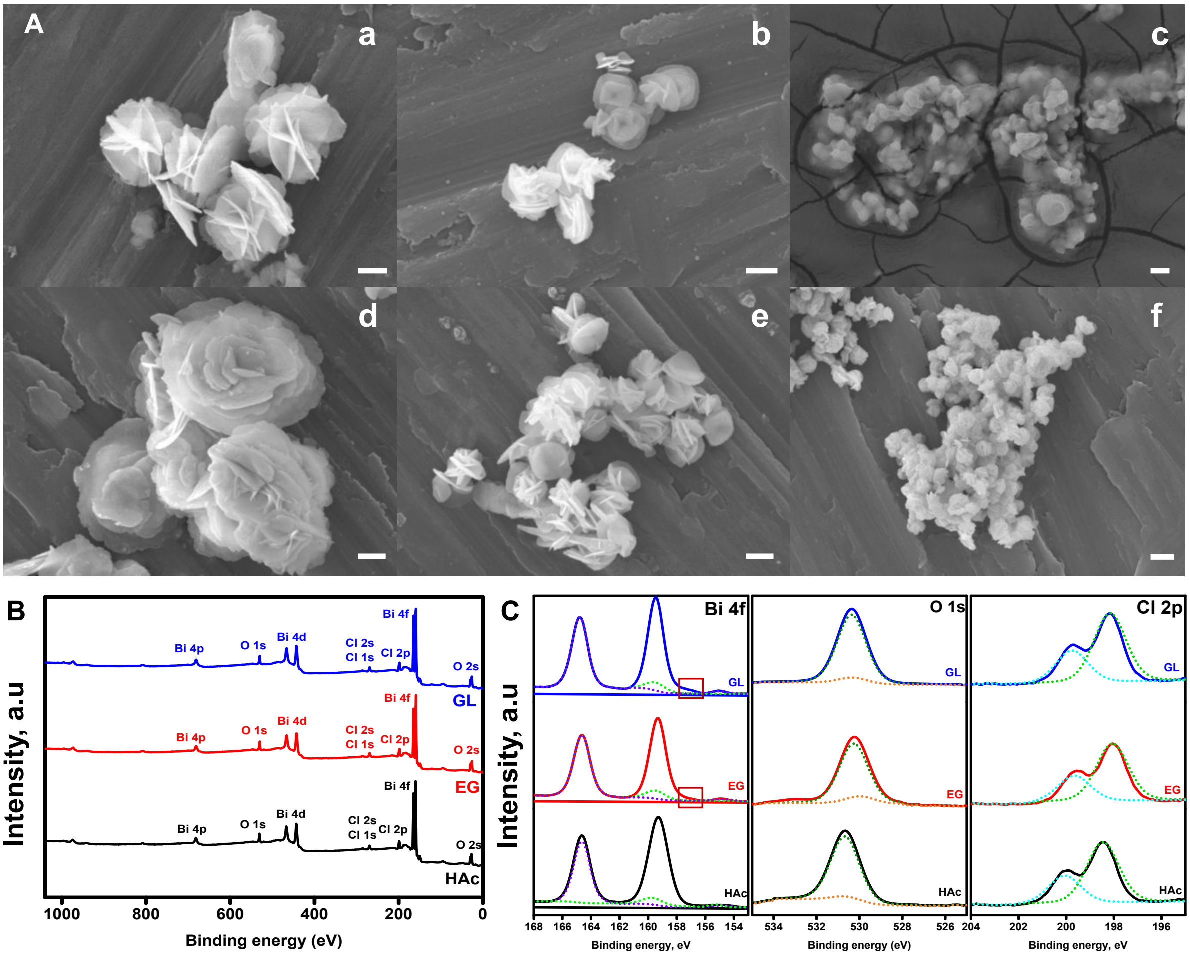

Figure S2. (A) SEM images showing the morphology of *E. coli*@Fe_3O_4@BiOCl micromotors and *S. aureus*@Fe_3O_4@BiOCl micromotors synthetized in the presence of HAc (a and d), EG (b and e) and GL (c and f). Scale bars, 1 µm. (B) XPS survey spectrum of *E. coli*@Fe_3O_4@BiOCl micromotors and (C) Bi 4f spectrum, O 1s spectrum, and Cl 2p spectrum.

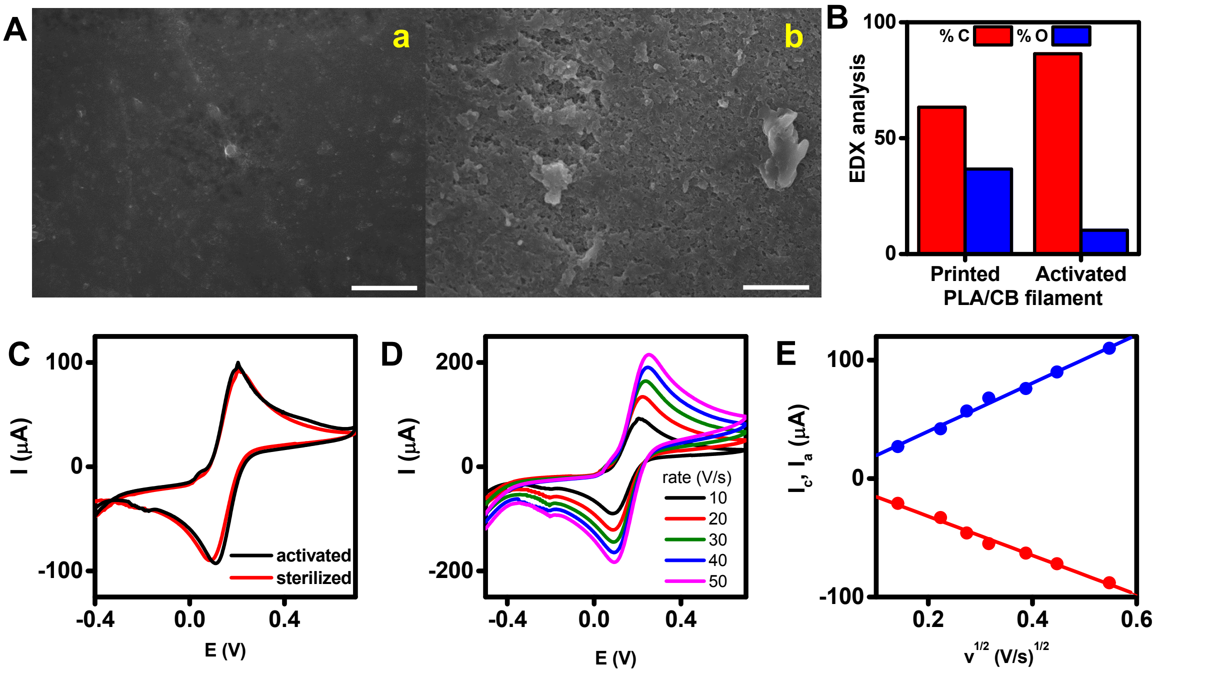

Fig. S3. (A) SEM images of the 3D-printed electrodes before (a) and after (b) activation and (B) corresponding EDX graph with the element composition. Such composition is expressed as a percentage based on the real surface area of each electrode. (C) Cyclic voltammograms in the presence of $[Fe(CN)_6]^{3-/4-}$ redox probe (10 mM in 0.1 M KNO_3) before (black) and after (red) the sterilization process, and (D) at different scan rates. (E) Linear variation of the current against the scan rate.

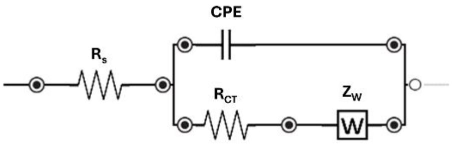

Fig. S4. Equivalent Randle's circuit (with a CPE element).

Table S1. Relation between the applied voltage and the magnetic field applied. Conditions: distance between the electromagnet and the micromotors: 3.5 cm. Angle of incidence: 90º

**Voltage (V)**	**Magnetic field on the electromagnet surface(G)**
**1.0**	4.10
**1.5**	5.99
**2.0**	8.80

Table S2. R_{CT} (kΩ·cm^2) values obtained from the fitting of EIS experimental using Nyquist equivalent circuit.

**BiOCl solvent crystallization**	**BiOCl**	***E. coli*@Fe_3O_4@BiOCl**	***S. aureus*@Fe_3O_4@BiOCl**
**HAc**	52±2	16±2	28±2
**EG**	58±2	21±2	43±2

E. coli@Fe_3O_4: 41±3 kΩ·cm
